# Supplementary material for: Folate deficiency disrupts key metabolic transitions within the developing neural ectoderm
Source: bioRxiv. 2026 Sep 22:2026.09.18.752622. Preprint. [Version 1] doi: 10.64898/2026.09.18.752622 (PMC13622060; doi:10.64898/2026.09.18.752622)
Supplement: Supplementary file 1 [file NIHPP2026.09.18.752622v1-supplement-5.pdf]

bioRxiv preprint doi: https://doi.org/10.64898/2026.09.18.752622; this version posted September 22, 2026. The copyright holder for this preprint (which was not certified by peer review) is the author/funder, who has granted bioRxiv a license to display the preprint in perpetuity. It is made available under a CC-BY 4.0 International license.

Dias et al., Figure S1

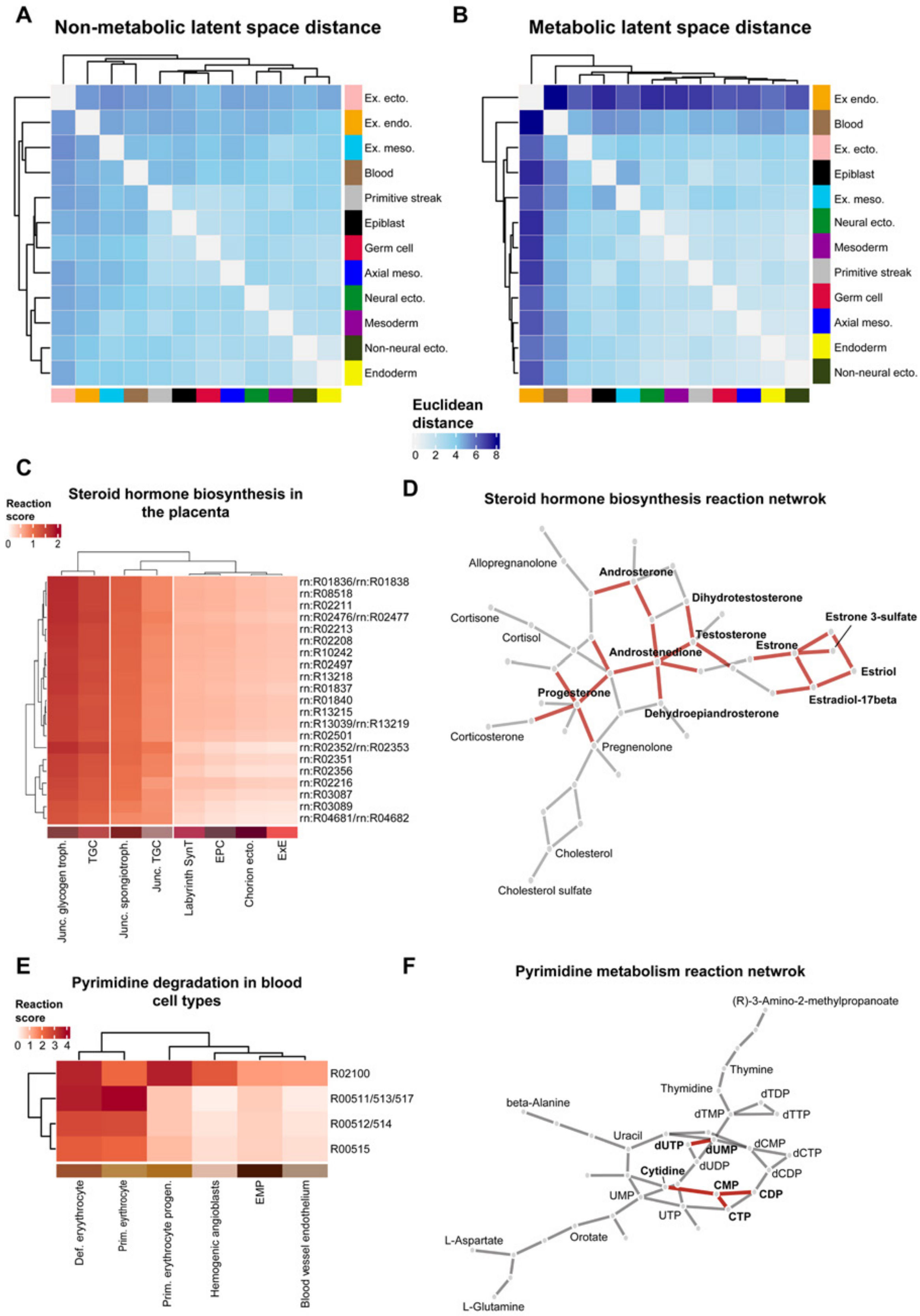

bioRxiv preprint doi: https://doi.org/10.64898/2026.09.18.752622; this version posted September 22, 2026. The copyright holder for this preprint (which was not certified by peer review) is the author/funder, who has granted bioRxiv a license to display the preprint in perpetuity. It is made available under a CC-BY 4.0 International license.

Figure S1. MeRN predicts metabolic reaction activity with cell-type resolution. A,B. Euclidean distance between lineages using average latent space positions for non-metabolic (**A**) and metabolic (**B**) predicted cell embeddings. **C**. Average extra-embryonic ectoderm reaction scores for selected reactions under the KEGG steroid hormone biosynthesis pathway, highlighting increased activity in the placental junctional zone compared to the labyrinth and chorion. Reactions were selected for variance > 0.2 within the extra-embryonic ectoderm. **D.** Network visualization of reactions in the KEGG steroid hormone biosynthesis pathway, showing the relationship between reactions (edges) and metabolites (nodes). Highlighted edges represent reactions displayed in (**C**). **E.** Average reaction scores for cell types in the blood and vascular cell lineage, highlighting activity of pyrimidine degradation reactions in differentiated erythrocytes. We curated reactions based on previously published descriptions of erythrocyte maturation[56,57]. **F.** Reaction network visualization for reactions in the KEGG pyrimidine metabolism pathway. Highlighted edges represent reactions displayed in (**E**).

bioRxiv preprint doi: https://doi.org/10.64898/2026.09.18.752622; this version posted September 22, 2026. The copyright holder for this preprint (which was not certified by peer review) is the author/funder, who has granted bioRxiv a license to display the preprint in perpetuity. It is made available under a CC-BY 4.0 International license.

Dias et al., Figure S2

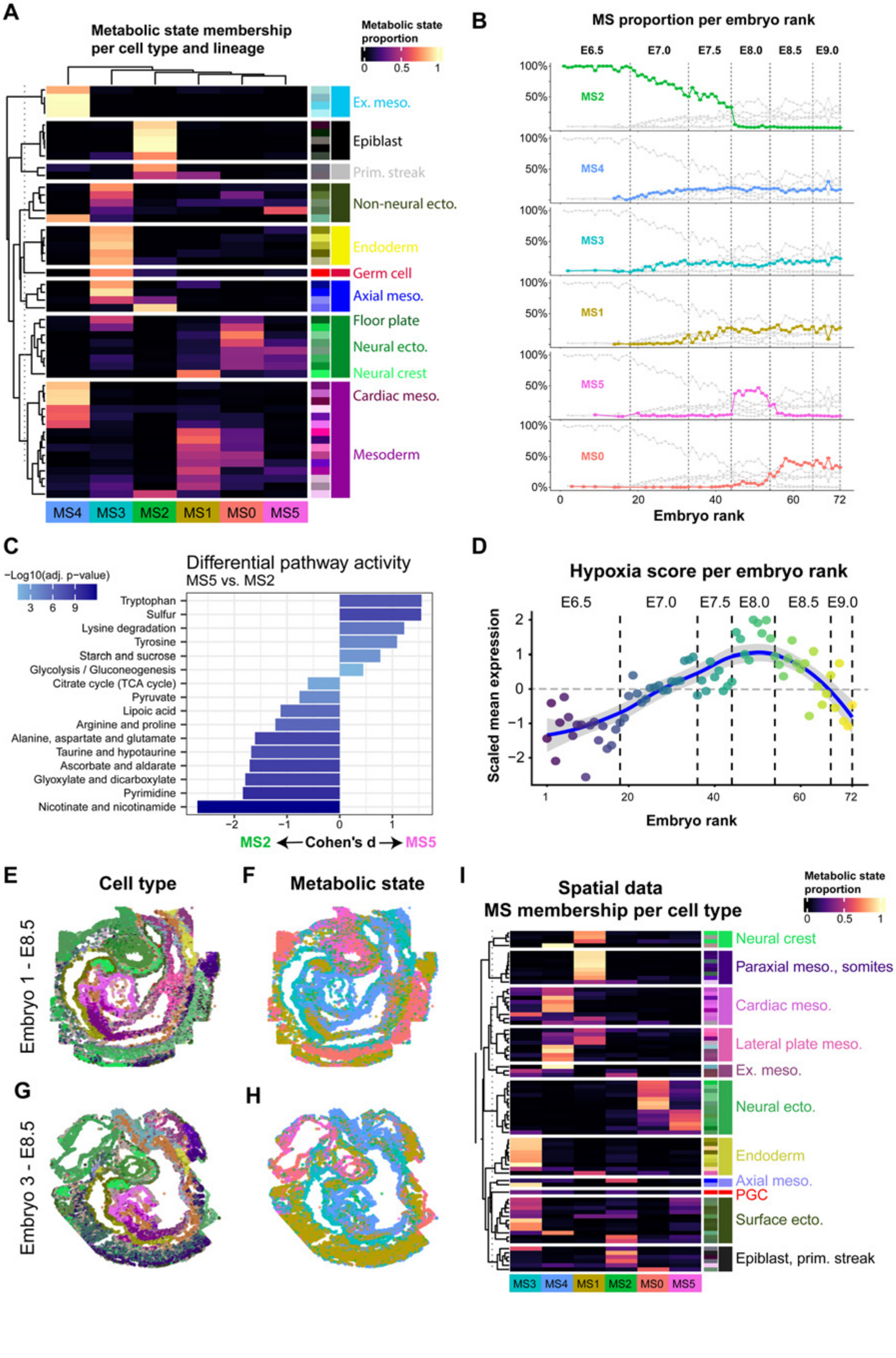

bioRxiv preprint doi: https://doi.org/10.64898/2026.09.18.752622; this version posted September 22, 2026. The copyright holder for this preprint (which was not certified by peer review) is the author/funder, who has granted bioRxiv a license to display the preprint in perpetuity. It is made available under a CC-BY 4.0 International license.

Figure S2. Temporal and spatial aspects of metabolic state. A. Heatmap of metabolic state assignments per cell type, showing the distribution of label assignments throughout the embryo. Row annotations represent individual cell types, split by overarching lineage, and columns represent metabolic states. Heat denotes the proportion of cells in a cell type assigned to each metabolic state; row values sum to 1 (100%). **B.** Proportion of metabolic state assignment per embryo, showing metabolic state abundance and dynamics over developmental time. Embryos were ranked from 1-72 according to transcriptional similarity to approximate a developmental continuum (see **Methods**). **C.** Effect size differences of preferential pathway usage between MS2 (E6.5-E7.5) and MS5 (E8.0). We curated KEGG pathways broadly related to redox homeostasis and energy metabolism that showed significantly different pathway activity ($p_{adj} < 0.05$ highlighting a preference for oxidative processes and redox protection in MS2 compared to MS5. **D.** Scaled mean expression of Hallmark hypoxia genes per embryo, displayed in ranked order as described in (**B**), highlighting maximal relative expression at E8.0, when MS5 is most abundant. **E,F,G,H.** Additional samples of seqFISH data of mouse embryos at E8.5, displayed in a sagittal section (replicates 1 and 3 from Harland et al.[75]). Cells are colored by cell type annotation (**E, G**) or predicted metabolic state (**F, H**). The spatial organization of metabolic states was consistent across all analyzed replicates. **I.** Heatmap of metabolic state assignments per cell type and lineage, as shown in (**A**), for all seqFISH data reported by Harland et al. Assignment patterns broadly matched our orthogonal data, including the association between early neural ectoderm and MS5, later neural ectoderm and MS0, cardiac mesoderm and MS4, and somitic mesoderm and MS1.

bioRxiv preprint doi: https://doi.org/10.64898/2026.09.18.752622; this version posted September 22, 2026. The copyright holder for this preprint (which was not certified by peer review) is the author/funder, who has granted bioRxiv a license to display the preprint in perpetuity. It is made available under a CC-BY 4.0 International license.

Dias et al., Figure S3

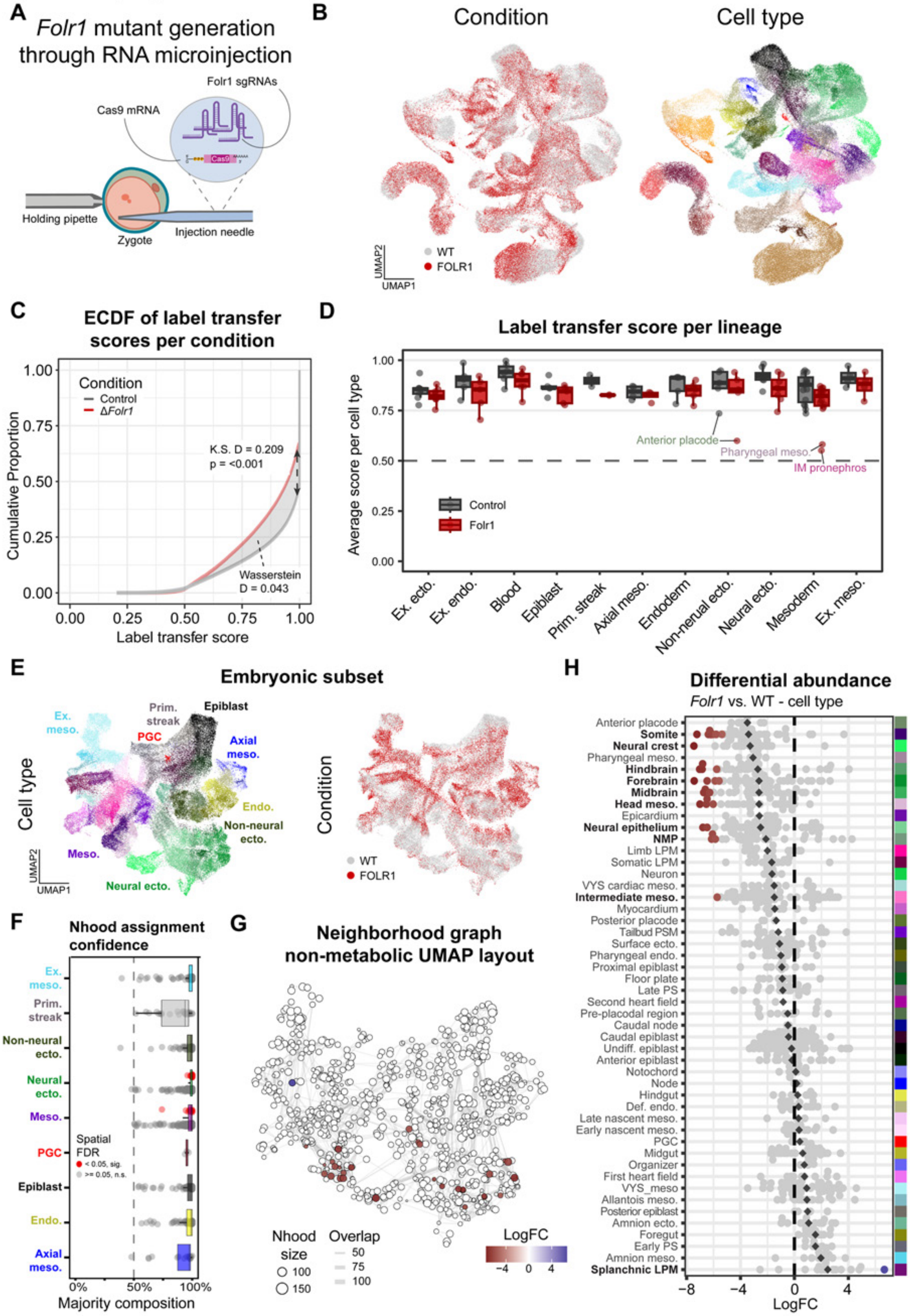

bioRxiv preprint doi: https://doi.org/10.64898/2026.09.18.752622; this version posted September 22, 2026. The copyright holder for this preprint (which was not certified by peer review) is the author/funder, who has granted bioRxiv a license to display the preprint in perpetuity. It is made available under a CC-BY 4.0 International license.

Figure S3. Generation and characterization of differential abundance in Δ*Folr1* embryos. **A.** Schematic of the CRISPR/Cas-9 method used to engineer zygotic crispant embryos lacking functional *Folr1*. **B.** Non-metabolic UMAP representation of the merged wild-type and Δ*Folr1* scRNA-seq data, colored by experimental condition (left) or cell type annotation (right). **C.** Empirical cumulative distribution function (ECDF) of label transfer scores, showing the difference in confidence for labels predicted for Δ*Folr1* (red) and wild-type (gray) cells, using the wild-type as a reference. Label transfer scores for each cell are cumulative across all possible cell-type labels and sum to 1.00 (100%). The gray area between curves represents the Wasserstein distance, and the double-sided arrow represents the Kolmogorov-Smirnov statistic, denoting the point of maximal vertical distance between distributions. **D.** Box plots of average label transfer score per lineage, representing confidence in label assignment. Each point represents a cell type, grouped by broad developmental lineages. Labeled points represent outliers with relatively low scores. **E.** Non-metabolic UMAP representation of the embryonic subset of merged Δ*Folr1* and wild-type cells, colored by cell type (left) and condition (right). This subset manually excludes cells in the extra-embryonic endoderm and ectoderm, as well as blood and vascular cell types. **F.** Boxplot of neighborhood composition per lineage, based on the K-nearest neighbors (KNN) graph calculated during differential abundance (DA) analysis. Points represent individual neighborhoods, positioned according to the proportion of cells belonging to the assigned label. Red points denote neighborhoods with significantly different Δ*Folr1*/wild-type ($\text{spatial FDR} < 0.05$). **G.** Non-metabolic UMAP layout of the KNN graph calculated using the non-metabolic latent space embeddings of wild-type and *Folr1* cells, highlighting neighborhoods with significant differential abundance and colored by log-fold-change (LogFC) calculated with Milo[87]. **H.** Differential abundance results using cell type labels, showing significant depletion of cell types in the neural ectoderm and trunk mesoderm, and enrichment of a single neighborhood in the splanchnic lateral plate mesoderm (LPM). Points represent individual neighborhoods; colored points represent significant neighborhoods, and color denotes LogFC, as in (**G**).

bioRxiv preprint doi: https://doi.org/10.64898/2026.09.18.752622; this version posted September 22, 2026. The copyright holder for this preprint (which was not certified by peer review) is the author/funder, who has granted bioRxiv a license to display the preprint in perpetuity. It is made available under a CC-BY 4.0 International license.

Dias et al., Figure S4

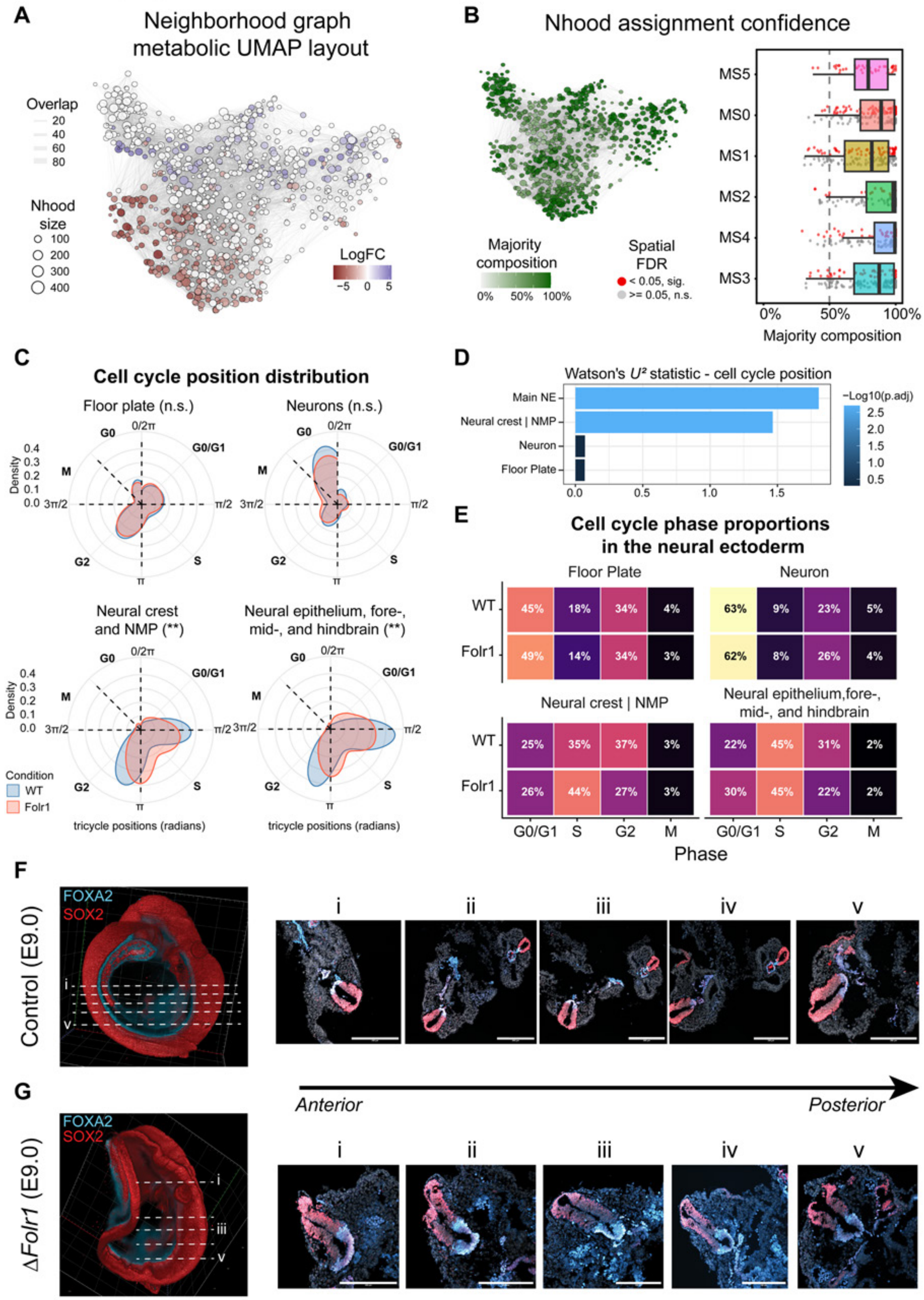

bioRxiv preprint doi: https://doi.org/10.64898/2026.09.18.752622; this version posted September 22, 2026. The copyright holder for this preprint (which was not certified by peer review) is the author/funder, who has granted bioRxiv a license to display the preprint in perpetuity. It is made available under a CC-BY 4.0 International license.

Figure S4. Cell depletion dynamics correlate with metabolic state and cell cycle dynamics. **A,B.** Metabolic UMAP layout of the KNN graph calculated using the metabolic latent space embeddings of wild-type and *Folr1* cells, highlighting neighborhoods with significant differential abundance and colored by LogFC calculated with Milo[87] (**A**) or proportion of cells in the assigned (majority) label (**B**). The box plot shows the proportion of cells in each neighborhood belonging to the assigned label, split by metabolic state. Colored points denote significant neighborhoods ($\text{spatial FDR} < 0.05$). **C,D.** Polar coordinate representation of neural ectoderm cell cycle position distributions calculated with tricycle[94], divided into four classes and displayed in (**C**). Significance was calculated using 999 permutations of Watson's two-sample test of homogeneity and adjusted with the Benjamini-Hochberg correction (**: $p_{adj} < 0.01$). Panel (**D**) also shows the adjusted p-value and Watson's U2 statistic per group. Main NE refers to the group containing neural epithelium, fore-, mid-, and hindbrain. **E.** Heatmap of cell cycle phase proportions per group, assigned according to cell cycle position, and highlighting the accumulation of neural crest and NMP cells in S phase, and main neural ectoderm cells in G0/G1 in response to genetic ablation of *Folr1*. **F,G.** Representative images of light-sheet microscopy sections of the neural tube, showing transverse sections of the neural tube and highlighting the expansion of the floor plate domain in Δ*Folr1* (**G**) compared to wild-type (**F**). Immunostaining shows markers for neural ectoderm (SOX2, red) and floor plate and axial mesoderm (FOXA2, blue). Dashed lines show the approximate location of the transverse sections, numbered in ascending order along the anterior-posterior axis. Scale bars: 200 μm.

bioRxiv preprint doi: https://doi.org/10.64898/2026.09.18.752622; this version posted September 22, 2026. The copyright holder for this preprint (which was not certified by peer review) is the author/funder, who has granted bioRxiv a license to display the preprint in perpetuity. It is made available under a CC-BY 4.0 International license.

Dias et al., Figure S5

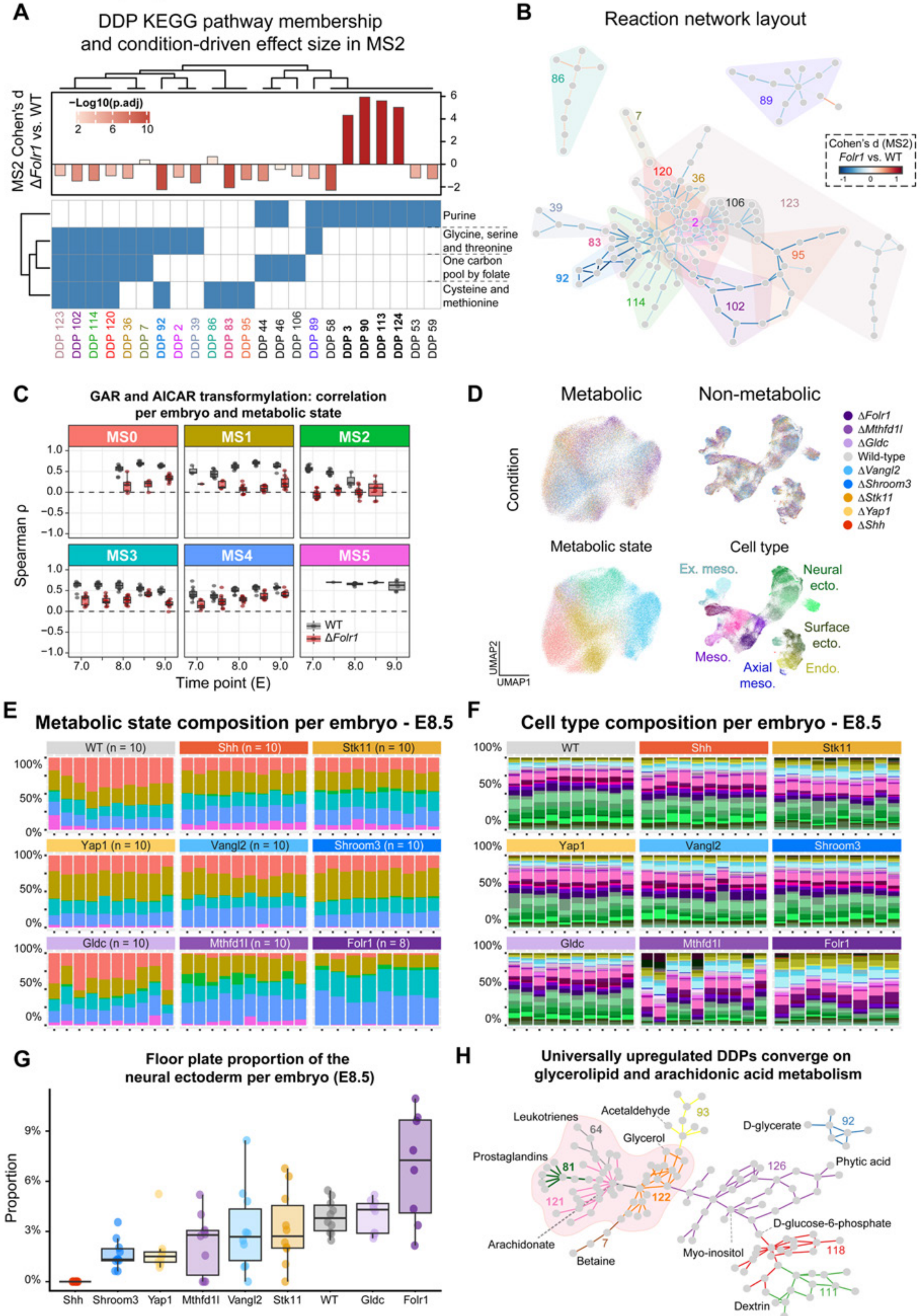

bioRxiv preprint doi: https://doi.org/10.64898/2026.09.18.752622; this version posted September 22, 2026. The copyright holder for this preprint (which was not certified by peer review) is the author/funder, who has granted bioRxiv a license to display the preprint in perpetuity. It is made available under a CC-BY 4.0 International license.

Figure S5. MeRN predicts metabolic determinants of neural tube defect etiology. A. Membership heatmap of DDPs in select folate-related KEGG pathways, highlighting the outsized effect of *Folr1* disruption on purine metabolism. **B.** Reaction network layout showing non-purine metabolism reactions displayed in (**A**), colored by reaction Cohen's d between WT and Folr1 in MS2. **C.** Box plots of Spearman correlation between GAR and AICAR transformylation reaction scores per embryo and metabolic state over time, indicating a global loss in correlation between folate-dependent reactions required for *de novo* purine biosynthesis. Each point represents an embryo; box plots are colored by condition. **D.** UMAP representations of the merged scRNA-seq data for 78 mutant embryos and 10 wild-type embryos at E8.5. UMAPs were generated using metabolic (left) or non-metabolic (right) latent space embeddings, and colored by experimental condition (top), metabolic state (bottom left), and cell type (bottom right). **E,F.** Bar plots of embryo composition. Each bar represents one embryo; slices are colored according to metabolic state (**E**) and cell type (**F**) labels, showing broad condition-driven compositional dynamics. **G.** Box plots denoting the proportion of the neural ectoderm represented by the floor plate, colored by experimental condition, and highlighting both the absence of the floor plate in Δ*Shh* crispant embryos and the enrichment of this ventral organizer in Δ*Folr1*. Points denote individual embryos. **H.** Reaction network layout showing the common group of DDPs upregulated across all mutant conditions (**64, 81, 121, 122**, highlighted). Notably, these DDPs are connected within glycerolipid and arachidonic acid metabolism, suggesting a common metabolic response to genetic insults associated with neural tube defects. The displayed DDPs include reactions within the glycerolipid, glycerophospholipid, or arachidonic acid metabolism KEGG pathways (**rn00561**, **rn00564**, and **rn00590**, respectively).

bioRxiv preprint doi: https://doi.org/10.64898/2026.09.18.752622; this version posted September 22, 2026. The copyright holder for this preprint (which was not certified by peer review) is the author/funder, who has granted bioRxiv a license to display the preprint in perpetuity. It is made available under a CC-BY 4.0 International license.

Dias et al., Figure S6

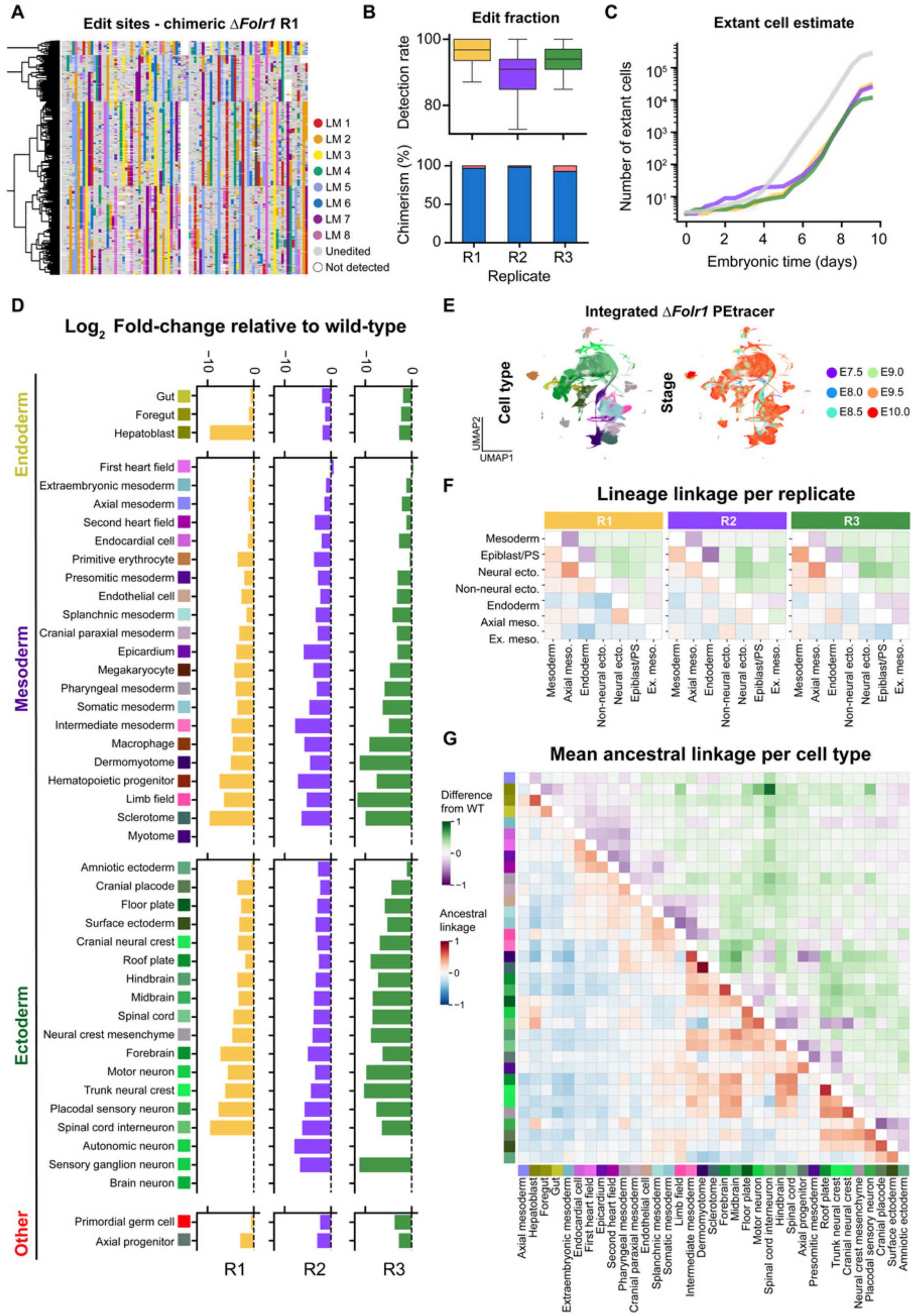

bioRxiv preprint doi: https://doi.org/10.64898/2026.09.18.752622; this version posted September 22, 2026. The copyright holder for this preprint (which was not certified by peer review) is the author/funder, who has granted bioRxiv a license to display the preprint in perpetuity. It is made available under a CC-BY 4.0 International license.

Figure S6. Chimeric Δ*Folr1* embryos display disrupted composition. A. Representative reconstructed lineage tree and character matrix for chimeric Δ*Folr1* replicate 1 (R1). Character matrix columns represent editable sites (3 per barcoded lineage-tracing cassette, 105 total sites) and are colored by each cell's edit state. LM = lineage marker. **B.** Box plots quantifying the per-cell detection rate for lineage-tracing cassettes (top) and bar plots denoting the proportion of the embryo represented by donor (blue) and host (pink) cells for each chimeric mutant replicate (bottom). R = Replicate. **C.** Estimated number of extant cells as a function of inferred developmental time based on branch length estimates, colored by replicate as in (**B**). Gray line represents the E9.5 reference mean. **D.** Δ*Folr1* $\log_2$ fold-change per cell type relative to the E9.5 reference, calculated per replicate chimeric mutant embryo. Missing bars denote complete absence in the Δ*Folr1* condition. **E.** UMAP representation of the integrated Δ*Folr1* and E7.5-E10.0 reference PEtracer data, colored by cell type as displayed in (**D**) (left) and by developmental stage. **F,G.** Mean pairwise ancestral linkage (**Methods**) at the level of broad lineages (**F**) and cell type (**G**). Below the diagonal (red-blue) indicates Δ*Folr1* linkage, with the mean across replicates in **G**, and above the diagonal (green-purple) indicates the relative difference from the mean E9.5 reference linkage between the same categories.
